# A novel nomogram and prognostic factor for metastatic renal cell carcinoma survival in the era of immune checkpoint inhibitors (ICIs)

**DOI:** 10.3389/fphar.2022.996404

**Published:** 2023-01-04

**Authors:** Mohammed Alradhi, Zewen Zhang, Mohammed Safi, Abdullah Al-danakh, Mokhtar Aldhbi, Salim Baldi, Li kui, Abdulaziz Alradhi, Saeed Bin Hamri, Ka Lun lo, Yi Zhao, Yang Jin

**Affiliations:** ^1^ Department of Urology, The Affiliated Hospital of Qingdao Binhai Univesity, Qingdao, China; ^2^ Department of Urology, Amran University, Amran, Yemen; ^3^ Department of Radiology, Jinan Central Hospital Affiliated to Shandong First Medical University, Jinan, China; ^4^ Department of Radiology, Shandong Provincial Hospital Affiliated to Shandong First Medical University, Jinan, China; ^5^ Department of Respiratory Diseases, Shandong Second Provincial General Hospital, Shandong University, Jinan, China; ^6^ Department of Urology, First Affiliated Hospital of Dalian Medical University, Dalian, China; ^7^ Research Center of Molecular Diagnostics and Sequencing, Axbio Biotechnology (Shenzhen) Co., Ltd., Shenzhen, China; ^8^ Department of Thoracic Surgery, Prince Mutaib Bin Abdulaziz Hospital, Al-jawf, Saudi Arabia; ^9^ Division of Urology, Department of Surgery, Ministry of the National Guard Health Affairs, Riyadh, Saudi Arabia; ^10^ Division of Urology, Department of Surgery, The Chinese University of Hong Kong, Hong Kong, Hong Kong SAR, China; ^11^ Department of Oncology, First Affiliated Hospital of Dalian Medical University, Dalian, China

**Keywords:** metastatic renal cell carcinoma, immune checkpoint inhibitors (ICI), nomogram, survival, SEER

## Abstract

Patients with metastatic renal cell cancer (mRCC) for whom surgery is ineffective may experience a poor prognosis. The different sites where cancer has spread, and the different ways to treat it in the immune checkpoint inhibitors era could help clinical decision-making. In this study, individuals with mRCC were selected from the SEER database between 2015 and 2016 based on the Food and Drug Administration (FDA) approval of ICIs. A total of 4011 mRCC patients were studied (2239 with lung metastasis vs. 797 with liver metastasis in the immune checkpoint inhibitors period). The age **≤** 64 years and male were the majority in all cases of mRCC. When the two groups (lung metastasis and liver metastasis) were compared, the liver metastasis group had more bone metastasis than the lung metastasis group (41.8% vs. 34.1%, *p* < 0.001), but the lung metastasis group had more brain metastasis (8.9% vs. 11.5%) (*p* = 0.023). In a study of overall survival (OS) in the ICI era for mRCC, we found that lung metastasis was significantly associated with improved survival compared to liver metastasis (*p* < 0.001: 7 months vs. 4 months). This survival advantage restricted in lung metastasis group of mRCC after adjusting age, sex, race, marital status, histological type, metastasis to bone, and brain, origin, radiotherapy record chemotherapy record, surgery on multivariable using Cox proportional hazard model (HR = 1.407; 95% CI = 1. 269−1.560; *p* < 0.001). The overall survival difference between the variables of the lung metastasis and liver metastasis was noted among most of the variables, with survival benefits restricted to patients in lung metastasis in the ICI era. Patients who had undergone chemotherapy and surgery were strongly positive predictors for better OS (HR = 0.427; 95% CI = 0.379−0.481; *p* < 0.001) (HR = 0.371; 95% CI = 0.311−0.444; *p*=< 0.001), and (HR = 0.313; 95% CI = 0.264−0.372; *p* < 0.001), (HR = 0.427; 95% CI = 0.320−0.568; *p* < 0.001) in lung metastasis group and liver metastasis group. The c-index of the prognostic nomogram for OS prediction was 0.74 and 0.73. This study found that patients with lung metastasis who received ICI had better survival than those with liver metastasis. Chemotherapy and surgery enhanced survival in kidney cancer patients, whereas radiation had little impact. We developed a complete and realistic nomogram for mRCC patients based on distant metastases to the lung and liver.

## 1 Introduction

RCC is one of the most common urinary system tumors, with clear cell carcinoma histology accounting for 60%–85% of all renal carcinomas. The global prevalence of kidney cancer has increased from around 200,000 new cases in 1990 to 393,000 in 2017, with almost 400,000 new cases found in 2018. At the current time, 20%–40% of people with a new diagnosis of RCC have metastatic disease (Siegel et al., 2022; Du et al., 2020; Strizova et al., 2019; Bossé et al., 2019; O'Connor et al., 2011). The great majority of patients present with conditions that can be surgically treated. However, more than one-third of persons treated with the hope of a cure will have a recurrence of metastatic disease; in RCC, lung metastasis are the most common, followed by bone, liver, and brain metastases (Bianchi et al., 2012a).

The treatment plans for patients with advanced-stage RCC have been difficult to implement. However, since 2011, FDA has approved several immunotherapeutic drugs for cancer, indicating that treatment options for patients with metastatic RCC have rapidly expanded over the last decade, with targeted ICI emerging as the new cornerstone treatment modality. Clinical studies are exploring many innovative ICI to examine if they might improve anti-tumor immune responses (Gong et al., 2016). The ICI that target the programmed cell death-1 (PD-1) and programmed cell death ligand-1 (PD-L1) axis have transformed the therapy landscape for RCC (Safi et al., 2021). The use of ICI for the treatment of ccRCC, either alone or in combination with other regimens, has recently advanced, and results have been encouraging. Currently, there have been four randomized trials that have investigated immune checkpoint inhibitors (ICIs) individually or in combination with other ICIs and VEGF/VEGF-R targeted drugs: ipilimumab and nivolumab in the clinical studies of CheckMate 214, or pembrolizumab and axtinib in other medical studies called KEYNOTE-426, as well as avelumab and axitaxitinib in JAVELIN Renal 101, and finally atezolizumab in combination with bevacizumab in IMmotion151, this demonstrates that it might be a feasible therapeutic option for those who experience recurrent RCC (Rini et al., 2019; Albiges et al., 2020; Leucht et al., 2022). Although these medicines are routinely used to treat advanced-stage RCC, no population-based study has been conducted to assess their survival advantages in patients with metastasis. We conducted this analysis using the (SEER-18) database to compare the OS rates of patients with mRCC in the ICI era.

## 2 Patients and methods

### 2.1 Data retrieval

The ethics statement permitted us to read the SEER research data files retrieved using the reference number 10237-Nov2018. The SEER database does not require informed patient consent for information distribution. SEER* Stat software was used to identify individuals with additional treatment data from SEER 18 registries in 2018. (version 8.3.9). As part of its mission, the National Cancer Institute’s (NCIs) SEER program collects and reports cancer incidence and survival data from a variety of sources. These figures are from central cancer registries in the United States, which cover roughly 30% of the population. SEER data includes patient demographics, initial tumor location, morphology, diagnosis stage, an initial course of cancer therapy, and vital status follow-up. We collected by selecting the primary site labeled of kidney site with years (2015−2016) which according to the FDA are treated with ICI and with only stage IV adult patients this data period is ICI era. The incidence statistics were combined with additional treatment fields to create this report. Only patients who had active follow-up during and after therapy were included in the study to reduce the missing data.

### 2.2 Prognostic description

**FIGURE 1:**
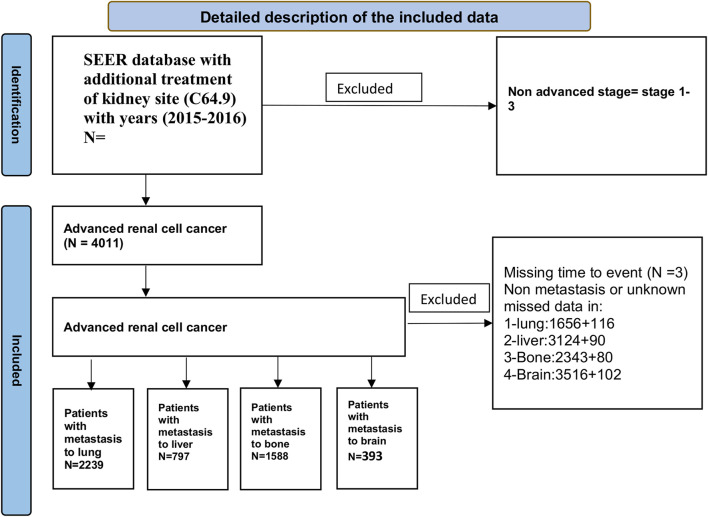
A detailed description of the mean deviation of the study.

The following variables were chosen for analysis: tumor subtype (based on the ICD-O-3 convention from the International Classification of Diseases for Oncology - Third Edition, considering only invasive tumors), histo-behavior (clear cell adenocarcinoma, papillary adenocarcinoma, others), metastatic sites (brain, bone, liver, or lung), laterality (left or right), age (20 years or more), sex (male or female), race (black, white (right, left, or others) taking part in radiation therapy, chemotherapy treatment, or surgical procedures, marital [yes or no (divorced, single, domestic, widowed, separated, unknown) ] and insurance statuses (yes or others). A detailed description is summarized in Figure 1. Our research’s final aim is OS, defined as the duration of time that individuals diagnosed with cancer have been alive since the date of diagnosis or the commencement of therapy.

### 2.3 Statistical analysis

#### 2.3.1 Clinical characteristics

The baseline demographic characteristics of patients were compared using the two-tailed test by the chi square for categorical variables with a *p*-value less than 0.05.

#### 2.3.2 The overall survival study

Kaplan-Meire analysis (K.M) to calculate the OS and log-rank tests were used to compare groups. The influence of a prognostic variable on OS was determined using univariate and multivariable Cox proportional hazards models with a *p*-value less than 0.05.

#### 2.3.3 The nomogram and clinical prediction

The nomogram was created as a predictor of survival for variables resulting from multivariable analysis. Finally, the parameters from the final model were utilized to create a nomogram and risk categorization system. Concordance index (c-index) is used to evaluate the nomogram model. C-index, a value range between 0 and 1, is to assess performance of the model. The receiver operating characteristic (ROC) curves are used to evaluate the nomogram’s ability to estimate patient mortality to that of the scoring criteria in lung metastasis and liver metastasis, respectively. All statistical analyses were done by SPSS version 26. Whereas nomogram and ROC were analyzed using the R program version 4.2.0 (http://www.r-project.org/).

## 3 Results

### 3.1 General patient’s characteristics

The majority of those diagnosed with mRCC were of the male gender in both lung and liver metastasis groups, respectively (68.6% vs. 65%). Metastatic to bone was significantly higher in the liver metastasis group than in the lung metastasis group (41.8% vs. 34.1%) (*p* = 0.001), while metastasis to the brain was significantly lower in the liver metastasis group than in the lung metastasis group (8.9% vs. 11.5%) (*p* = 0.023). All characteristics are summarized in full in Table 1, and Supplementary Table S1.

**TABLE 1:** Demographic characteristics of advance RCC lung metastasis vs. liver metastasis in the Unites State based on SEER database.

Parameters	Lung mets	Liver mets	*p*-Value
Age			
≤64	1184 (52.8)	386 (48.4)	
>65	1056 (47.2)	412 (51.6)	<0.001
Sex			
Male	1537 (68.6)	518 (65.0)	
Female	702 (31.4)	279 (35.0)	0.03
Race			
White	1843 (82.3)	634 (79.5)	
Black	223 (10.0)	115 (14.4)	
Others	173 (7.7)	48 (6.0)	0.01
Marital status			
Yes	1294 (57.8)	446 (56.0)	
No	945 (42.2)	351 (44.0)	0.196
Origin			
Left	1074 (48.0)	386 (48.4)	
Right	1083 (44.4)	375 (47.1)	
Others	82 (3.7)	36 (4.5)	0.511
Grade			
I-II	108 (9.3)	50 (9.4)	
III - IV	797 (35.6)	235 (29.5)	
Unknown	1234 (55.1)	512 (64.2)	<0.001
Histology			
Clear cell adenocarcinoma	1708 (76.3	535 (67.1)	
Papillary adenocarcinoma	95 (4.2)	38 (4.8)	
Others(including unspecified renal cell carcinoma)	436 (19.5)	224 (28.1)	<0.001
Bone mets			
Yes	763 (34.1)	333 (41.8)	
No	1476 (65.9)	464 (58.2)	<0.001
Brain mets			
Yes	258 (11.5)	71 (8.9)	
No	1981 (88.5)	726 (91.1)	0.023
Radiation status			
Yes	526 (23.5)	170 (21.3)	
No	1713 (76.5)	627 (78.7)	0.115
Chemotherapy			
Yes	1301 (58.1)	428 (53.7)	
No	938 (41.9)	369 (46.3)	0.017
Surgery			
Yes	788 (35.2)	181 (22.7)	
No	1451 (64.8)	616 (77.3)	<0.001

**FIGURE 2:**
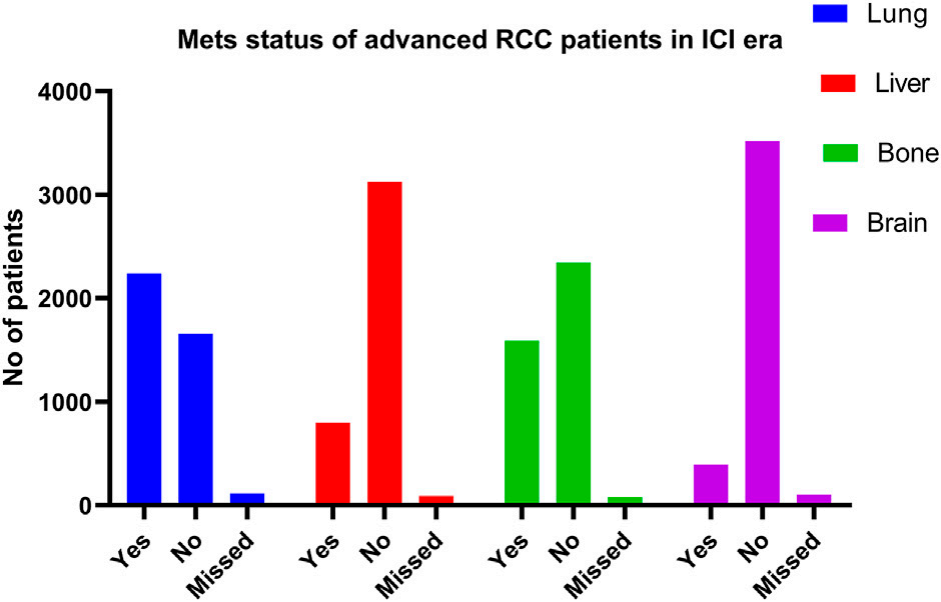
By K.M of metastasis sites of advanced RCC in ICI era.

**FIGURE 3:**
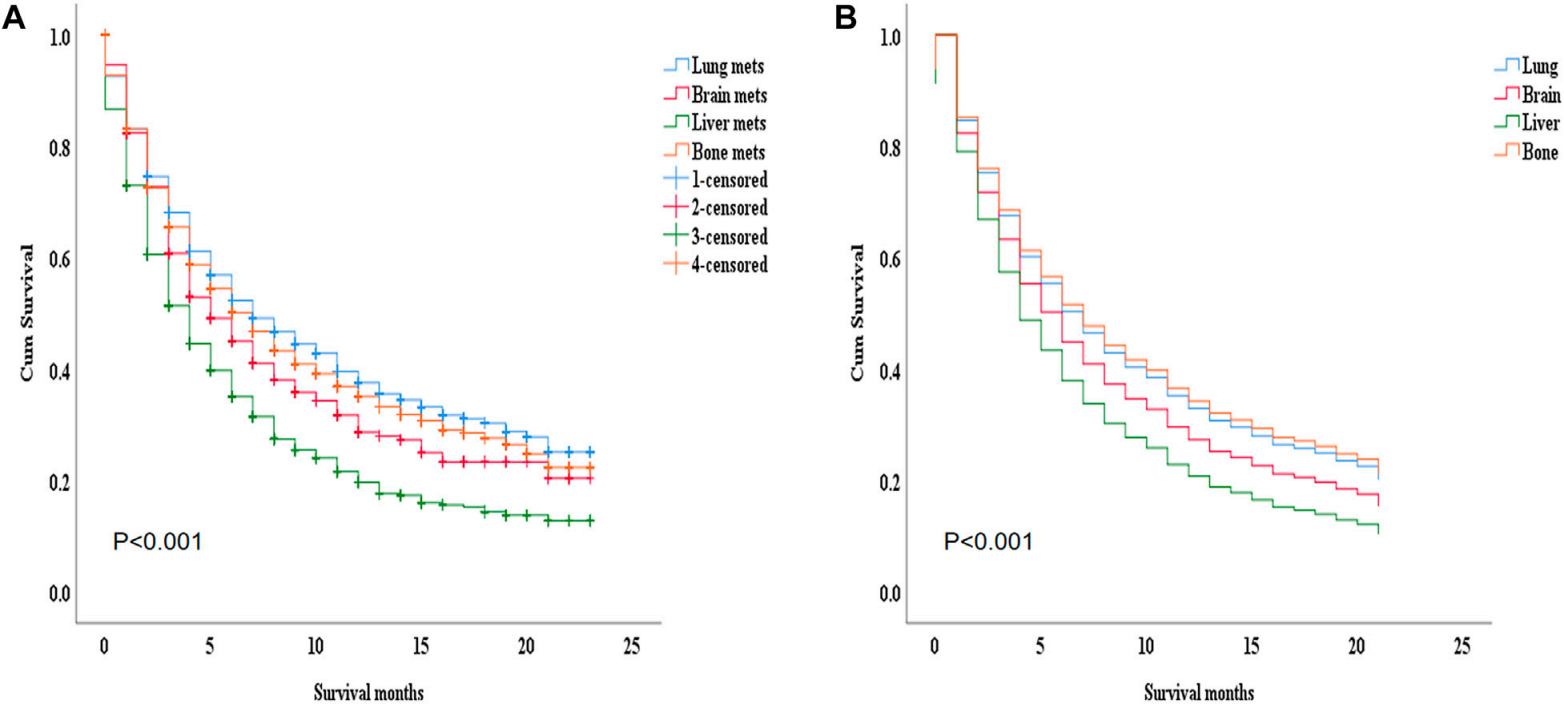
**(A)** The K.M study of the metastasis site in the advanced RCC to brain, bone, lung, and liver we found that the liver got the worst survival among them, **(B)** even after adjusting the variables on multivariable using Cox proportional hazard model. *p*=< 0.001.

The SEER database has 4011 cases of mRCC with 2239 lung metastasis, 797 liver metastasis, 1588 bone metastasis, and 393 brain metastasis in the ICI era Figure 2. In the K.M examination of metastasis sites in mRCC to brain, bone, lung, and liver, we observed that the liver had the lowest survival among them, even after adjusting the variables on multivariable using the Cox proportional hazard model (*p* < 0.001), as results our study comprehensively focused on the lung and liver metastasis groups. Figure 3.

### 3.2 The overall survival study

**FIGURE 4:**
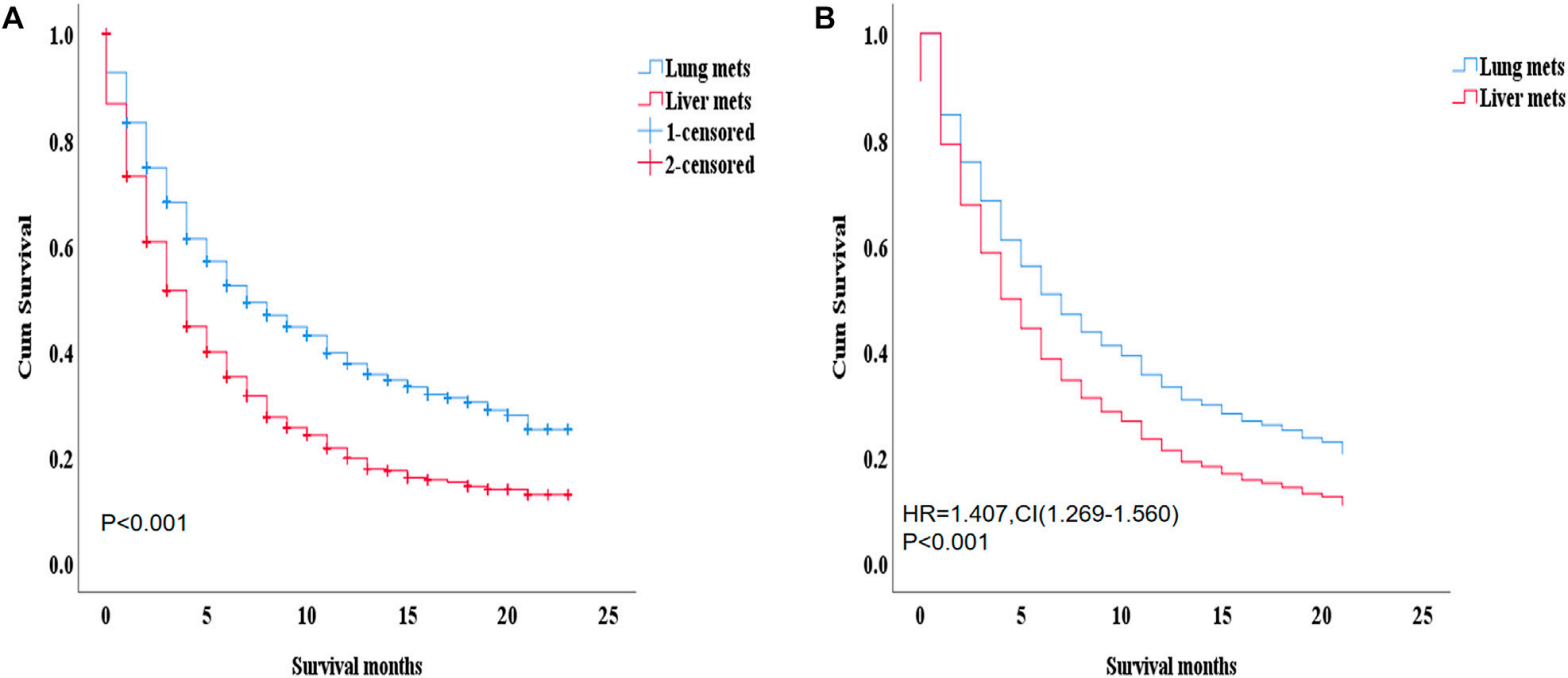
**(A)** by K.M with median survival (*p* =< 0.001: 7 months vs. 4 months). This survival advantage restricted in patients of the lung metastasis **(B)** after adjusting age, sex, race, marital status, histological type, metastasis to (bone, brain), origin, radiotherapy record chemotherapy record, surgery on multivariable using Cox proportional hazard model (HR = 1.407; 95% CI = 1. 269−1.560; *p*=< 0.001).

**FIGURE 5:**
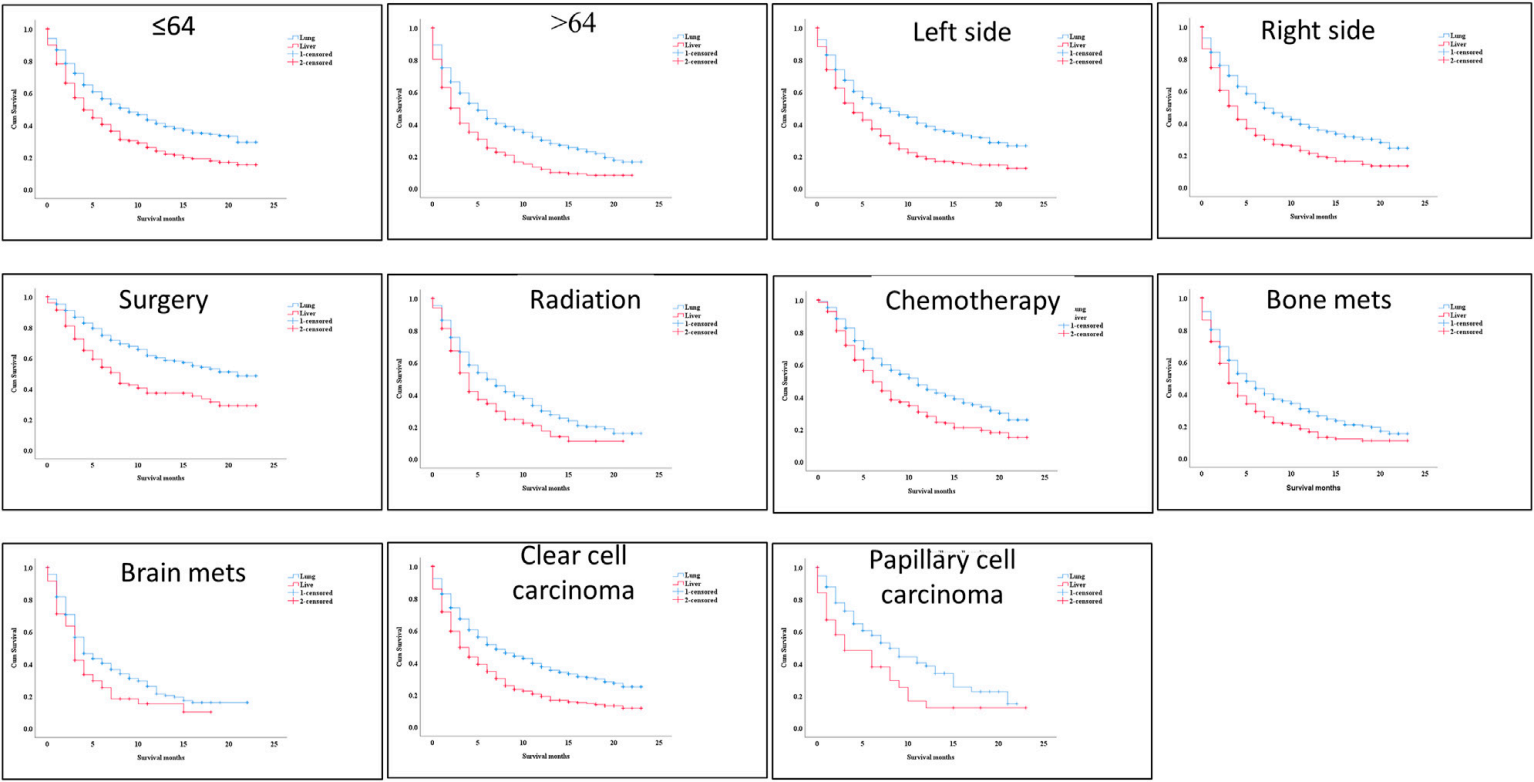
K–M between the two groups and the better with survival benefits restricted to patients in lung metastasis in the immunotherapy era, such as younger age (≤64), clear cell carcinoma, Papillary cell carcinoma, radiotherapy status, chemotherapy, and surgery showed a highly significant difference in OS median (9 months vs. 5 months, 7 months vs. 3 months, 6 months vs. 4 months, 11 months vs. 6 months, and 21 months vs. 8 months, *p*=< 0.001) respectively.

During the ICI era, we noticed that mRCC patients with lung metastasis had a much longer median survival time than those with liver metastasis (7 months vs. 4 months; *p* < 0.001). Figure 4A. Even after we adjusted the two groups with the variables for age, gender, race, marital status, histological type, metastasis to (bone, brain), origin, radiotherapy record, chemotherapy record, and surgery using a Cox proportional hazard model the mRCC who had metastasis to the liver showed poor survival compared to the lung metastasis group (HR = 1.407; 95% CI = 1.269−1.560; *p* < 0.001). Figure 4B. In the K.M study of mRCC, the difference in OS between the variables of the lung metastasis and liver metastasis were noted among most of the variables, with survival benefits restricted to patients in lung metastasis in the ICI era, such as younger age (≤64), clear cell carcinoma, papillary cell carcinoma, radiotherapy status, chemotherapy, and surgery showed a highly significant difference in OS median (9 months vs. 4 months, 7 months vs. 3 months, 6 months vs. 4 months, 11 months vs. 6 months, and 21 months vs. 8 months; *p* < 0.001) respectively Figure 5 Table 2.

**TABLE 2:** OS study using Kaplan-Meier analysis between the variables in the Unites State based on SEER database.

Variables	Median months lung mets 7	Median months liver mets 4	*p*-Value
Age (years) at diagnosis			
≤64	9	5	<0.001
>65	6	4	<0.001
Race			
white	7	4	<0.001
Black	5	4	0.284
Others	10	3	<0.001
Gander			
female	7	4	<0.001
Male	8	4	<0.001
Marital status			
Yes	8	4	<0.001
No	6	4	<0.001
Origin			
Left	8	4	<0.001
Right	7	4	<0.001
Others	4	1	<0.001
Grade			
Well, moderate differentiated	18	11	0.11
Poorly differentiated, Undifferentiated	11	5	<0.001
Unknown	5	3	<0.001
Histology			
Clear cell adenocarcinoma	7	3	<0.001
Papillary adenocarcinoma	8	3	0.016
Others(including unspecified renal cell carcinoma)	8	4	<0.001
Mets to bone			
No/unknown	9	4	<0.001
Yes	5	3	<0.001
Mets to brain			
No	8	4	<0.001
Yes	4	3	0.021
Surgery			
No/unknown	4	3	<0.001
Yes	21	8	<0.001
Chemotherapy			
No/unknown	3	1	<0.001
Yes	11	6	<0.001
Radiotherapy			
No/unknown	8	4	<0.001
Yes	6	4	<0.001
Marital status			
Married	16	11	0.005
Not married	25	15	0.001

**FIGURE 6:**
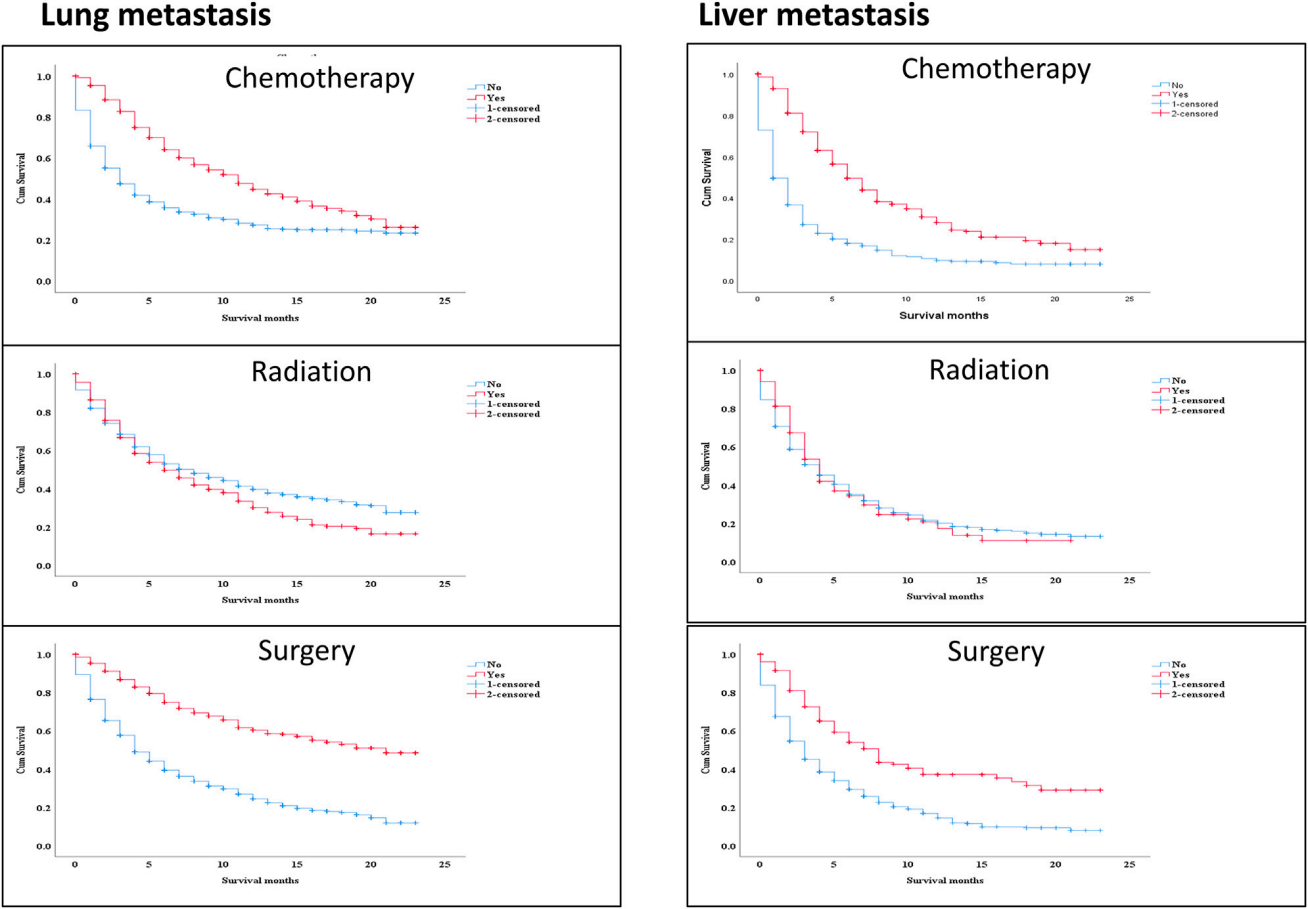
The survival effect of some variables inside lung metastasis group and liver metastasis group revealed better survival in the treatment modalities (chemotherapy and surgery). Interestingly, radiotherapy, there was no difference in survival between treating with radiation or not in the liver metastasis group.

The survival effect of several factors within the lung metastasis and liver metastasis groups demonstrated improved survival in treatment methods (chemotherapy and surgery). Surprisingly, there was no difference in survival in the liver metastasis group between those who received radiation and those who did not Figure 6 Multivariable analysis of the ICI era showed that radiotherapy treatment in distant-stage RCC was the independent predictor of worse survival in both groups. While, patients who had undergone chemotherapy and surgery were strongly positive predictors for better OS (HR = 0.427; 95% CI = 0.379−0.481; *p* < 0.001), (HR = 0.371; 95% CI = 0.311−0.444; *p*=< 0.001), and (HR = 0.313; 95% CI = 0.264−0.372; *p* < 0.001), (HR = 0.427; 95% CI = 0.320−0.568; *p* < 0.001) in both groups (lung metastasis and liver metastasis) Table 3.

**TABLE 3:** Univariate and multivariable analyses of advance RCC lung metastasis vs. liver metastasis in the Unites State based on SEER database.

Parameters	Lung mets				Liver mets			
	Univariate HR/CI	*p*-value	Multivariate HR/CI	*p*-value	Univariate HR/CI	*p*-value	Multivariate HR (95% CI)	*p*-value
Age (≤64 vs. >64	1.501 (1.301−1.642)	<0.001	1.251 (1.111−1.460)	<0.001	1.495 (1.281−1.77)	<0.001	1.126 (0.943−1.338)	0.190
Sex male vs. female	0.916 (0.811−1.034)	0.154	-	-	0.943 (0.792−1.122)	0.508	-	-
Race								
White	Reference	0.004	Reference	0.233	Reference	0.715	-	-
Black	1.290 (1.081−1.540)	0.005	1.008 (0.837−1.214)	0.932	0.929 (0.664−1.299)	0.665	-	-
Others	0.850 (0.683−1.057)	0.144	0.827 (0.664−1.031)	0.091	0.855 (0.574−1.272)	0.439	-	-
Marital yes vs. no	0.785 (0.700−0.880)	<0.001	0.947 (0.841−1.066)	0.365	1.011 (0.854−1.196)	0.902	1.127 (0.949−1.338)	0.173
Origin								
Left	Reference	0.001	Reference	0.728	Reference	0.391	-	-
Right	0.997 (0.887−1.119)	0.955	0.981 (0.873−1.102	0.741	1.034 (0.872−1.227)	0.699	-	-
Other	1.643 (1.254−2.154)	<0.001	1.093 (0.831−1.438)	0.525	1.381 (0.887−1.958)	0.171	-	-
Grade								
i–ii	Reference	<0.001	Reference	<0.001	Reference	<0.001	Reference	<0.001
iii–iv	0.432 (0.343−0.544)	<0.001	1.919 (1.496−2.461)	<0.001	0.409 (0.272−0.614)	<0.001	2.499 (1.614−3.869	<0.001
Unknown	0.542 (0.477−0.614	<0.001	1.697 (1.340−2.149)	<0.001	0.713 (0.590−0.862)	<0.001	1.157 (0.912−1.469)	0.230
Histology/behav								
Clear cell adenocarcinoma	Reference	0.725	-	-	Reference	0.450	-	-
Papillary adenocarcinoma	0.982 (0.743−1.298)	0.897	-	-	1.129 (0.933−1.368)	0.213	-	-
Others (including unspecified renal cell carcinoma)	0.941 (0.812−1.091)	0.423	-	-	1.137 (0.754−1.715)	0.541	-	-
Mets at bone yes vs. others	1.436 (1.278.-1.613)	<0.001	1.319 (1.156−1.506)	<0.001	1.198 (1.012−1.417)	0.035	0.872 (0.734−1.036)	0.119
Mets at brain yes vs. others	1.414 (1.197−1.670)	<0.001	1.480 (1.221−1.795)	<0.001	1.202 (0.905−1.596)	0.205	-	-
Radiotherapy yes vs. no	1.170 (1.028−1.333)	0.018	0.919 (0.779−1.085)	0.319	0.971 (0.791−1.191)	0.775	-	-
Chemotherapy yes vs. no	0.483 (0.431−0.542)	<0.001	0.427 (0.379−0.481)	<0.001	0.401 (0.339−0.467)	<0.001	0.371 (0.311−0.444)	<0.001
Surgery yes vs. others	0.335 (0.292−0.385)	<0.001	0.3130.264−0.372)	<0.001	0.485 (0.387−0.606)	<0.001	0.427 (0.320−0.568)	<0.001

### 3.3 The nomogram and clinical prediction

**FIGURE 7:**
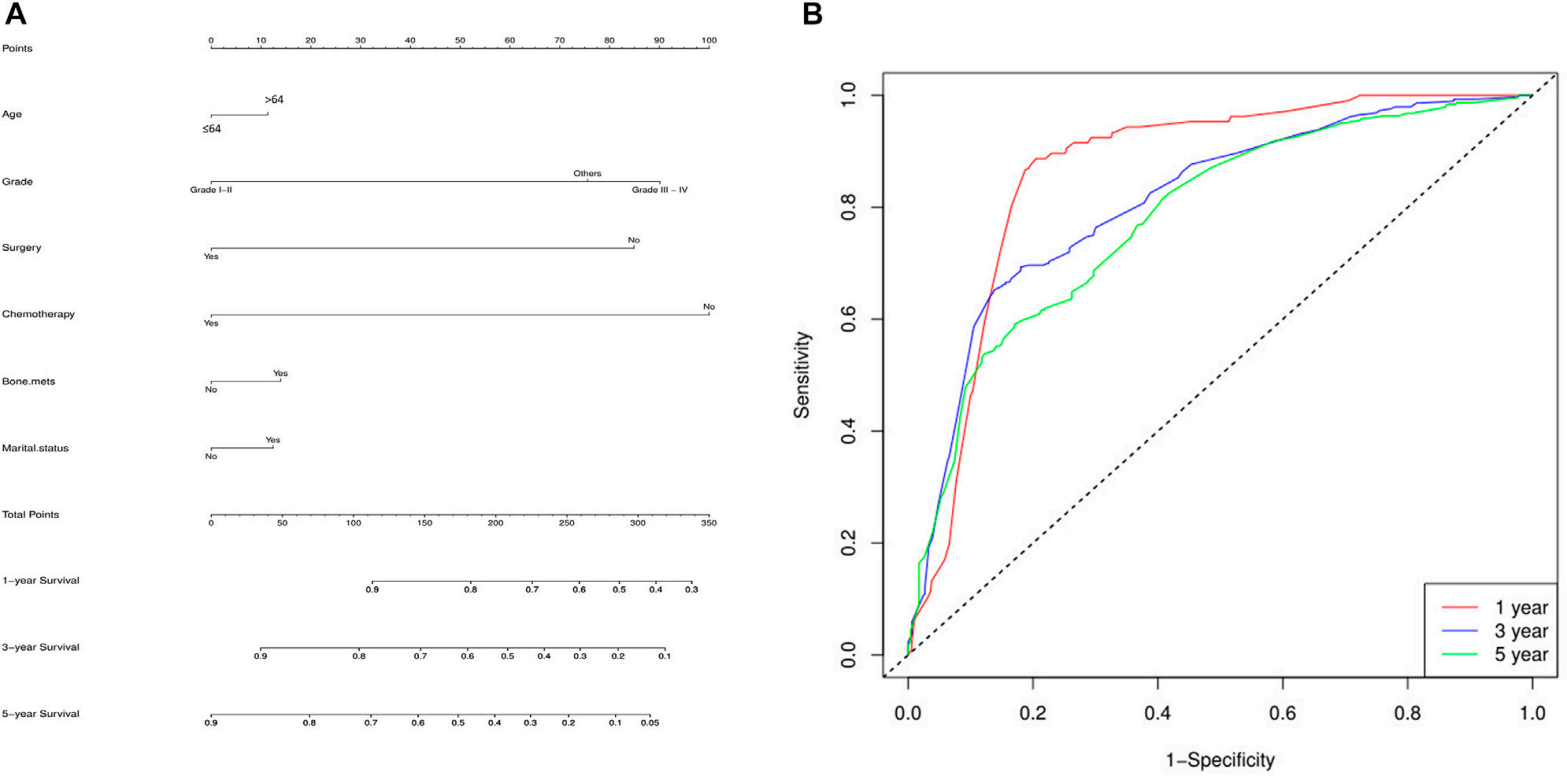
Lung metastasis group, **(A)** the c-index of the prognostic nomogram for OS prediction was 0.74. The prediction model revealed that the most critical factor influencing prognosis was surgery, followed by chemotherapy. **(B)** To evaluate the nomogram’s performance, 1-, 3-, and 5-year receiver operating characteristic (ROC) curves were created. The assessment revealed significantly improved prediction accuracy. AUC values of 0.87, 0.82, and 0.80 were obtained for the 1-, 3-, and 5-year survival nomograms, respectively.

**FIGURE 8:**
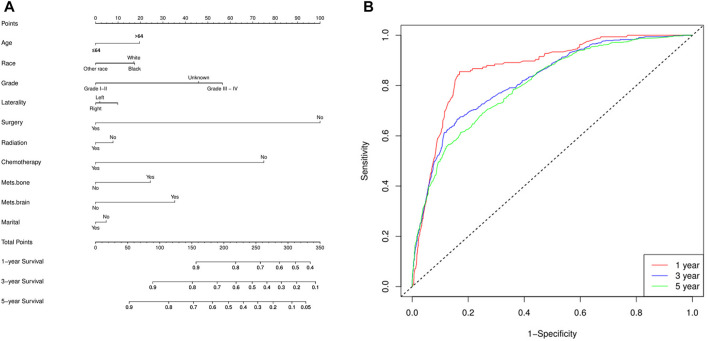
The liver metastasis group **(A)** the c-index of the prognostic nomogram for OS prediction was 0.73, and **(B)** the nomogram’s performance, 1-, 3-, and 5-year receiver operating characteristic (ROC). AUC values of 0.86, 0.81, and 0.73 were obtained for the 1-, 3-, and 5-year survival nomograms, respectively.

Finally, a nomogram model was built based on the prognostic factors that were significantly positive predictors of survival multivariable cox regression of mRCC of the two groups (lung metastasis and liver metastasis) separately. Each prognostic parameter was assigned a score based on its prognostic value, and the sum total of the scores was used to predict 1-3 and 5-year survival. The sum of the scores for all factors was turned into an estimate of the likelihood of death in the experiment. In the lung metastasis group, the c-index of the prognostic nomogram for OS prediction was 0.74. The prediction model revealed that the most critical factor influencing prognosis was surgery, followed by chemotherapy. To evaluate the nomogram’s performance, 1-, 3-, and 5-year receiver operating characteristic (ROC) curves were created. The assessment revealed significantly improved prediction accuracy. AUC values of 0.87, 0.82, and 0.80 were obtained for the 1-, 3-, and 5-year survival nomograms, respectively Figure 7. On the other hand, in the liver metastasis group, the c-index of the prognostic nomogram for OS prediction was 0.73. AUC values were (0.86, 0.81, and 0.73) in 1, 3, and 5 years respectively, Figure 8.

## 4 Discussion

Globally, the prevalence of mRCC is increasing. Although most cases are detected early enough for surgical excision to be curative, up to one-third of individuals have recurrence of tumors (Kim et al., 2021). Additionally, around 15% of RCC patients have metastasize to other regions of the body, rendering surgery futile. Clinical, laboratory, and radiographic manifestations of mRCC all have an impact on the disease’s natural history, which may range from several months to several years, depending on the disease’s characteristics. Cancer of the kidneys is a wide word that refers to a number of diverse entities, each with its own set of molecular abnormalities that set it apart from the others. Compared to other RCC varieties, the most prevalent variety is characterized by its angiogenic and immunogenic tumor microenvironment (TME), which is defined by intricate interactions between the stroma and the immune system. TME heterogeneity, like tumor cell heterogeneity, manifests as a varied distribution and phenotype, increasing the risk that a tumour may resist therapy (Deleuze et al., 2020). Before the introduction of immunological and targeted therapy, mRCC was a difficult disease to treat with a poor prognosis. ICI for RCC has grown into a routine aspect of the treatment regimen, including monoclonal antibodies directed against the antigens programmed cell death 1 (PD-1) and cytotoxic T lymphocyte antigen 4 (CTLA-4). Some fascinating findings have been reported from the development of ICI, either alone or in combination with other drugs (Motzer et al., 2019; Motzer et al., 2020). Patients with RCC may benefit from targeted ICI in addition to antiangiogenic therapy since this highly vascularized tumor is also an immunogenic tumor with a substantial number of immune cells such as tumor-infiltrating lymphocytes (Thompson et al., 2007b; Leite et al., 2015). In the first research, Thompson et al. employed immunohistochemistry to analyze PD-L1 expression in RCC. They observed that 24% of staining was linked to negative pathologic characteristics such as advanced tumor stage, increased tumor size, Fuhrman nuclear grade 3 or 4, and tumor necrosis (Thompson et al., 2007a). All of this measure a significant gain in survival for a subset of people. Moreover, the anti-PD-1 monoclonal antibody nivolumab displayed a greater objective response rate and longer overall survival in patients with recurrent RCC compared to everolimus (Motzer et al., 2015; Alradhi et al., 2022). Our study was prompted by information on good results from clinical trials, including targeted therapies. Our study is the largest in which of the number of patients with mRCC metastases to the lung and liver treated with ICI and found a substantial survival advantage. Previous research indicated that in the absence of ICI, metastases to the lung, bone, liver, and brain occurred at rates of 54.9%, 37.7%, 19.5%, and 10.4%, respectively (Bianchi et al., 2012a). This rate was comparable to what we saw throughout the ICI era, which was the subject of our research (Chandrasekar et al., 2017). Metastatic site variation in relation to treatment modalities may help clinical decision-making by providing appropriate data sources. While liver metastasis have been shown in patients and experimental models to restrict ICI efficacy, a combination of liver-directed radiation and ICI has been shown in patients to increase systemic anti-tumor immunity (Golden et al., 2013; Formenti et al., 2018; Yu et al., 2021). It is still unclear which metastatic sites should be targeted with radiation to maximize immune-stimulatory effects when combined with ICI. In our study, we observed that the liver metastasis group had worse survival than the lung metastasis group and that most of the factors in the liver metastasis group had lower OS. Jiali and colleagues discovered that liver metastases were related with a worse OS in melanoma patients, regardless of tumor load, number of metastatic sites, age, gender, prior lines of therapy, or blood lactate dehydrogenase (LDH) levels. Furthermore, the researchers confirmed that individuals with melanoma who only had liver involvement benefited less from ICI than those with lung involvement (Yu et al., 2021). Moreover, colorectal and gastric cancer studies reveal that individuals with lung or liver metastasis are more likely to develop bone metastasis than those without lung or liver metastasis (Qiu et al., 2018). When the percentage of black persons with liver metastasis was compared to that of other races, it was shown that they had a higher prevalence of the disease. Regardless of marital status, men had a greater risk of liver metastasis than women, and married patients had a higher percentage of metastasis than unmarried individuals. Depending on the conditions, the data collected in our study may help clinicians with diagnosis, prognosis, and other aspects of each individual patient. Our study resulted in the development of a comprehensive and practical nomogram for estimating the 1-, 3-, and 5-year prognosis of mRCC patients, which takes into account both clinical and pathological factors. When a mathematical model is combined with a large number of relevant parameters to forecast a future endpoint, the result is visually represented as an easy-to-use nomogram. Because metastasis has such a large influence on prognosis in the ICI era, the current study aimed to create a comprehensive and practical nomogram for predicting the survival probability of mRCC patients in the clinic based on distant metastatic to lung and liver. Several mRCC nomograms have been developed in order to predict recurrence and OS in mRCC patients. Kattan et al. developed the model in 2001 to predict the chance of recurrence in mRCC patients after surgery. Chinese researchers studied nephrectomy patients with clear cell renal cell carcinoma and created a nomogram to predict both overall and disease-specific survival in this patient population. For patients who had routine clear cell mRCC surgery, Sorbellini and colleagues developed a postoperative prognostic nomogram to aid the prediction of recurrence in patients with mRCC (Kattan et al., 2001; Sorbellini et al., 2005; Zhang et al., 2018). In the treatment modalities, chemotherapy and surgery used as a monotherapy in mRCC improve survival relative to non-use, whereas, there is no change in radiation treatment, and that could be due to the significant impact of newly discovered targeted treatments in recent years (Al-Danakh et al., 2022). In the study of chemotherapy and surgery usage, better survival significance were connected to therapeutic applications of both chemotherapy and surgery. In overall multivariable analysis confirms that OS in mRCC patients with lung metastasis was better than any metastatic groups (brain, bone, liver) in the ICI era. Some of our study’s limitations were the prevalence of comorbidities and the absence of performance status information. Furthermore, the study excluded a detailed explanation of patients’ ICI and missed the geographical region details. Moreover, it is unknown which treatments the patients underwent as each treatment modifies survival time in its own unique way. To our knowledge, this is the biggest population-based study to date evaluating the survival benefit of checkpoint inhibitors in mRCC with metastasis sites.

## 5 Conclusion

According to the findings of this study, mRCC individuals with lung metastasis those received ICI had higher survival than those with liver metastasis. Chemotherapy and surgery improved OS in patients with metastatic stage kidney cancer. Radiation, on the other hand, had minimal effect. Considering that distant metastasis to the lung and liver has such a significant impact on prognosis in the ICI era, the goal of this study was to develop a comprehensive and practical nomogram for predicting the chance of survival in RCC patients based on distant metastasis to the lung and liver. Extensive study and large cohort data are necessary in order to better identify the OS difference across metastatic locations and establish possible treatment targets.

## Data Availability

The datasets presented in this study can be found in online repositories. The names of the repository/repositories and accession number(s) can be found below: https://seer.cancer.gov/.
