# Supplementary material for: A novel nomogram and prognostic factor for metastatic renal cell carcinoma survival in the era of immune checkpoint inhibitors (ICIs)
Source: Front Pharmacol. 2023 Jan 4;13:996404. doi: 10.3389/fphar.2022.996404 (PMC9846485; doi:10.3389/fphar.2022.996404)
Supplement: Supplementary file 1 [file Table1.docx]

Supplementary table 1

| **Parameters** | **Bone mets(%)** | **Brain mets(%)** |
| --- | --- | --- |
| **Age** |  |  |
| **≤64** | 739(52.8) | 234(59.5) |
| **>65** | 849(47.2) | 159(40.5) |
| **Sex** |  |  |
| **Male** | 1076(67.7) | 281(71.5) |
| **Female** | 512(32.3) | 112(28.5) |
| **Race** |  |  |
| **White** | 1321(83.1) | 329(83.7) |
| **Black** | 167(10.5) | 30(7.6) |
| **Others** | 100(6.2) | 34(8.6) |
| **Marital status** |  |  |
| **Yes** | 915(57.7) | 220(55.9) |
| **No** | 673(42.3) | 173(44.1) |
| **Origin** |  |  |
| **Left** | 756(47.6) | 183(46.5) |
| **Right** | 750(47.2) | 193(49.1) |
| **Others** | 82(5.2) | 17(4.3) |
| **Grade** |  |  |
| **I-II** | 129(8.2) | 43(11) |
| **III - IV** | 446(28.1) | 106(27) |
| **Unknown** | 1013(63.7) | 244(62.0) |
| **Histology** |  |  |
| **Clear cell adenocarcinoma** | 1241(78.1) | 348(88.5) |
| **Papillary adenocarcinoma** | 75(4.7) | 10(2.6) |
| **Others(including unspecified renal cell carcinoma)** | 341(17.2) | 35(8.9) |
| **Liver mets** |  |  |
| **Yes** | 333(21) | 71(17.1) |
| **No** | 1226(77.2) | 322(81.9) |
| **Unknown** | 29(1.8) | 0 |
| **Lung mets** |  |  |
| **Yes** | 763(48) | 258(65.6) |
| **No** | 783(49.4) | 135(34.4) |
| **Unknown** | 42(2.6) | 0 |
| **Brain mets** |  |  |
| **yes** | 172(10.8) | - |
| **No** | 1384(87.2) | - |
| **Unknown** | 32(2) |  |
| **Bone mets** |  |  |
| **yes** | - | 172(43.7) |
| **No** | - | 221(56.3) |
| **Radiation status** |  |  |
| **Yes** | 849(53.4) | 291(74.1) |
| **No** | 739(46.5) | 102 (25.9) |
| **Chemotherapy** |  |  |
| **Yes** | 915(57.6) | 227(57.7) |
| **No** | 673(42.3) | 166(42.3) |
| **Surgery** |  |  |
| **yes** | 1156(72.7) | 295(76) |
| **No** | 432 (27.3) | 98(24) |
